# Reproductive and Obstetric Outcomes Following a Natural Cycle vs. Artificial Endometrial Preparation for Frozen–Thawed Embryo Transfer: A Retrospective Cohort Study

**DOI:** 10.3390/jcm12124032

**Published:** 2023-06-13

**Authors:** Andrea Roberto Carosso, Nicole Brunod, Claudia Filippini, Alberto Revelli, Bernadette Evangelisti, Stefano Cosma, Fulvio Borella, Stefano Canosa, Chiara Benedetto, Gianluca Gennarelli

**Affiliations:** 1 Obstetrics and Gynecology 1U, Physiopathology of Reproduction and IVF Unit, Department of Surgical Sciences, Sant’Anna Hospital, University of Torino, 10124 Turin, Italy; 2 Department of Surgical Sciences, Clinical Statistics, University of Torino, 10124 Turin, Italy; 3 Obstetrics and Gynecology 2U, Department of Surgical Sciences, Sant’Anna Hospital, University of Torino, 10124 Turin, Italy

**Keywords:** frozen embryo transfer (FET), obstetric outcome, endometrial preparation, natural cycle

## Abstract

**Background:** The proportion of frozen embryo transfer cycles has consistently grown in recent decades. Some adverse obstetric outcomes after frozen embryo transfer could possibly be explained by different approaches in endometrial preparation. The aim of the present study was to investigate reproductive and obstetric outcomes after frozen embryo transfer, comparing different endometrial preparation strategies. **Methods:** This retrospective study included 317 frozen embryo transfer cycles, of which 239 had a natural or modified natural cycle and 78 underwent artificial endometrial preparation. After excluding late abortion and twin pregnancies, the outcomes of 103 pregnancies were analyzed, 75 of which were achieved after a natural cycle/modified natural cycle, and 28 were achieved after an artificial cycle. **Results:** The overall clinical pregnancy rate/embryo transfer was 39.7%, the miscarriage rate was 10.1%, and the live birth rate/embryo transfer was 32.8%, without significant differences in reproductive outcomes between natural/modified cycle and artificial cycle groups. The risks of pregnancy-induced hypertension and abnormal placental insertion were significantly increased in pregnancies achieved after the artificial preparation of the endometrium (*p* = 0.0327 and =0.0191, respectively). **Conclusions:** Our study encourages the use of a natural cycle or modified natural cycle for endometrial preparation for frozen embryo transfer in order to ensure the presence of a corpus luteum able to orchestrate maternal adaptation to pregnancy.

## 1. Introduction

The proportion of ART cycles with frozen/thawed embryo transfers (FETs) has been rising steadily over the last decade. According to ESHRE data, FETs accounted for approximately 20% of all in vitro fertilization (IVF) cycles performed in Europe in 2010, and this figure increased to 40% in 2018 [1]. The popularity of FET has increased since the procedure contributes significantly to cumulative live birth rates while simultaneously allowing for single embryo transfers, virtually eliminating the chances of twin pregnancies [2]. Furthermore, the possibility of freezing all embryos (the freeze-all strategy) is associated with a dramatic reduction in the incidence of severe ovarian hyperstimulation syndrome (sOHSS) [3]. 

Of note, the potential benefits of FET also extend to maternal and fetal outcomes: the risks of preterm birth and low birth weight (LBW) and small for gestational age (SGA) newborns are reduced following FET when compared to the outcomes of fresh ET [4]. 

On the other hand, FET has been associated with increased risks of some pregnancy complications and abnormal neonatal outcomes, such as macrosomia and large for gestational age (LGA) babies, hypertensive disorders of pregnancy (HDP), preterm premature rupture of membranes (pPROM), anomalies of placental insertion, and a need for a Cesarean section (CS) [5,6]. It is suggested that these adverse outcomes could be partially explained by the type of endometrial preparation for FET. However, only a few studies have addressed this issue thus far. 

The most common strategies currently used for endometrial preparation are represented by either natural cycles (NCs)/modified natural cycles (m-NCs) or artificial cycles (ACs). In the NC protocol, the timing of ET is established according to the urinary preovulatory LH peak detected in a spontaneous ovarian cycle, while in the m-NC protocol, a human chorionic gonadotropin (hCG) trigger is administered when a preovulatory follicle is identified during an ultrasound examination. In the AC protocol, the endometrium is prepared via the administration of exogenous estrogens and progesterone [2,7]. In the latter case, ovulation does not occur; hence, a corpus luteum (CL) is lacking. Recent data support the notion that the absence of a CL could be the main culprit for the increased incidence of obstetric complications [8,9,10]; indeed, factors normally produced by the CL are involved in the physiology of uterine and systemic adaptation to pregnancy [11].

The aim of the present study was to investigate reproductive and obstetric outcomes after FET, comparing different endometrial preparation strategies with or without the presence of a CL.

## 2. Materials and Methods

A retrospective, real-life cohort study was carried out at the Sant’Anna Academic Hospital. Overall, 317 patients undergoing FET were included. Seventy-eight FETs were performed after AC, and 239 FETs were performed after NCs/m-NCs. 

Twin pregnancies, late abortions, and therapeutic terminations of pregnancy were excluded from the statistical analyses. For patients selected for NC/m-NC preparation, ultrasound investigations were scheduled between days 7 and 10 of a spontaneous cycle. When the endometrial thickness reached 7 mm and a dominant follicle was identified (a follicle with a diameter of 14 mm), the patients began to take urinary LH peak detection tests (Clearblue^®^) on a daily basis. In selected cases, such as ultrasound evidence of a pre-ovulatory follicle before a positive LH test could be detected or patients with negative LH tests in previous cycles despite the presence of a follicle upon ultrasound monitoring, ovulation was triggered via subcutaneous hCG injection (5000 IU). The trigger was administered when a single follicle reached a mean diameter of least 18 mm. The ET was performed on day +6 from the urinary LH surge (NC) or on day +7 from the hCG trigger (m-NC). A soft Guardia catheter (Cook Ltd., Limerick (Ireland)) was used under US control as previously described [12]. In all cases, the luteal phase was supported by 180 mg/day intravaginal natural progesterone (Crinone 8, Merck, Darmstadt (Germany)), which was initiated three days before the ET. 

In the AC protocol, the endometrium was prepared by administering transdermal estradiol (Dermestril^®^) 100 mcg/48 h (150 in overweight/obese patients), beginning on the 1st day of menstruation and increasing the dose up to 200 mcg/48 h in the case of an insufficient response. Once the endometrial thickness reached at least 7 mm, a priming dose of 5000 IU hCG was administered subcutaneously. The next day, 180 mg/day of intravaginal natural progesterone (Crinone 8, Merck, Darmstadt (Germany)) was initiated. In the case of a positive pregnancy test, the estradiol and progesterone were continued until 12 weeks of gestational age.

The reproductive outcomes considered in the study were the clinical pregnancy rate per ET (cPR/ET), miscarriage rate per ET, and live birth rate per ET (LBR/ET). A clinical pregnancy was defined as the presence of an embryo with cardiac activity at an ultrasound examination performed 15–20 days after a positive pregnancy test. The miscarriage rate was defined as the proportion of patients with a clinical pregnancy who spontaneously miscarried before a gestational age of 12 weeks. The live birth rate was defined as the proportion of patients who delivered a live infant (>24 weeks). 

The obstetric maternal outcomes were the gestational age at delivery, delivery mode (spontaneous, operative, or Cesarean section (CS)), postpartum hemorrhage (PPH, defined as blood loss > 500 mL after a vaginal birth and >1000 mL after a CS), grade 3 or 4 perineal tears (obstetrical anal sphincter injuries, OASIS), hypertensive disorders of pregnancy (HDPs), pregnancy-induced hypertension (PIH), gestational diabetes (GDM), preterm premature rupture of membranes (pPROM), chorioamniotitis, gestational cholestasis, placental anomalies (abnormal localization/insertion; placental retention after birth), need of hospitalization during pregnancy, and need of labor induction. 

The following neonatal outcomes were considered: late preterm birth (32–37 weeks), early preterm birth (<32 weeks), birthweight, small for gestational age (SGA, <10° centile considering gestational age and sex), large for gestational age (LGA, >90° centile considering gestational age and sex), early and late intrauterine growth restrictions (IUGRs, before 32 weeks and between 32 and 37 weeks, respectively), neonatal macrosomia (birthweight > 4000 g), APGAR score at 5′ < 7, and Neonatal Intensive Care Unit (NICU) hospitalization. 

The variables relating to the patients’ characteristics and hormonal treatments were: age at the time of embryo cryopreservation, age at ET, body mass index (BMI) at ET, smoking habit, cause of infertility (when multiple causes of infertility were reported, the definition “mixed infertility” was adopted), the presence of polycystic ovary syndrome (PCOS), antral follicular count (AFC), anti-Müllerian hormone (AMH) circulating level, the presence of comorbidities and/or previous gynecological surgery, oocyte fertilization technique (conventional IVF or ICSI), embryo cryopreservation technique (slow-freezing or vitrification), endometrial thickness at ET, the number of transferred embryos, and low-dose acetylsalicylic acid (ASA) prophylaxis during pregnancy.

The results of descriptive statistics were expressed as absolute numbers (*n*) or percentages (%) for categorical variables, while continuous variables were reported as means and standard deviations (SDs). Gestational age was reported via the median and interquartile range (IQR). Either Student’s t-test or the Wilcoxon–Mann–Whitney test were used to compare continuous variables, according to the type of distribution. The Chi-squared test and Fischer’s exact test were used to compare categorical variables. Odds ratios (ORs), expressed with a confidence interval of 95%, were estimated in a univariate analysis to assess the effects of endometrial preparation (NC/m-NC vs. AC) on maternal and perinatal outcomes. The same analyses were performed while adjusting for selected co-variates (maternal age > 40 years, maternal BMI > 30, the presence of PCOS, ICSI, and ASA prophylaxis for maternal outcomes; the child’s sex, HDP, and GDM for neonatal outcomes). Statistical analyses were performed using the SPSS Statistics software, version 28.0.1.0, and SAS for Windows, version 9.4. A *p* value < 0.05 was considered statistically significant.

## 3. Results

A total of 317 FET cycles were analyzed, 239 of which occurred with NC/m-NC endometrial preparation and 78 with the AC protocol (Table 1). Overall, 143 positive pregnancy tests were obtained. A total of 16 first-trimester miscarriages and 4 ectopic pregnancies were registered, and 107 pregnancies evolved beyond 12 weeks of gestation. Among these pregnancies, two were terminated in the second trimester after a diagnosis of abnormal fetal karyotype, one was spontaneously aborted at 14 weeks, and one was excluded from the analysis as it was a twin pregnancy. Eventually, the statistical analysis included 103 pregnancies, 75 of which were obtained after NC/m-NC endometrial preparation and 28 after the AC protocol.

- Patient characteristics and treatment variables:

A comparison between the NC/m-NC and AC groups of pregnant women is reported in Table 2. The groups were comparable with respect to maternal age, ethnicity, BMI, and smoking habit, whereas they differed in the duration of infertility, which was significantly longer in the NC/m-NC group. More women in the AC group were diagnosed with anovulatory infertility and with PCOS. Both AFCs and AMH levels were significantly higher than in the NC/m-NC group. The freeze-all strategy, which is usually utilized to prevent severe OHSS, was more frequent in the AC group.

- Reproductive outcomes:

The overall clinical pregnancy rate/ET was 39.7%, the miscarriage rate was 10.1% (*n* = 32), and the overall live birth rate/ET was 32.8% (*n* = 104, of which one was a twin birth). No statistically significant differences in reproductive outcomes were registered between the two groups (Table 2).

- Obstetric maternal outcomes:

The risks of PIH, abnormal placental insertion, and PPH were significantly increased in the pregnancies obtained via the AC protocol (Table 3). There were no differences regarding gestational age, type of delivery, incidence of GDM, cholestasis, HDP, PE, pPROM, or chorioamniotitis (Table 3).

A univariate logistic regression analysis showed an increased risk of PIH among women undergoing the AC protocol (OR = 3.94; IC 95%, 1.492–10.389). This result was confirmed after adjusting for the selected confounders (aOR = 4.38; IC 95%, 1.507–12.747) (Table 4). Due to the small number of observations, the OR for abnormal placental insertion was not calculated.

- Neonatal outcomes:

No statistically significant differences were observed between the two study groups. No cases of early preterm delivery or early IUGR (<32 weeks) were reported. No relationship was observed between the type of endometrial preparation and relevant neonatal outcomes (Table 5).

## 4. Discussion

The most relevant finding of the present study was that the incidences of both PIH and PPH were significantly higher when the endometrium was prepared with exogenous estradiol and progesterone than when ET was performed during a spontaneous ovulatory cycle. As a matter of fact, the risk of PPH was more than doubled and the risk of PIH was four times higher in the AC group of patients.

These results, which are in line with those from very recent reports [6,13,14], were obtained from a limited series of observations. This makes the clinical findings even more striking, considering that virtually only pregnancies from single-blastocyst transfers were included (in total, only three double-blastocyst transfers were performed, resulting in one twin pregnancy, which was excluded from the analysis) and that an adjustment for several relevant confounders was performed. 

Our results and the recent results of others might have interesting implications for clinical practice. 

As a matter of fact, while the number of IVF cycles is constantly increasing worldwide [15], the proportion of FETs is rising even more rapidly. This is mainly due to the improvement of cryopreservation techniques. As an example, the strategy of freezing all embryos (the freeze-all strategy) is becoming increasing popular since it ensures a live birth rate at least equal to that of fresh ET while virtually eliminating the risk of ovarian hyperstimulation syndrome [16]. 

For this reason, in recent years, the focus has been increasingly placed on pregnancy outcomes in terms of complications for both the mother and the offspring over the potential impact of the type of endometrial preparation on FET success rates. 

In 2017, a Cochrane review of studies on pregnancy and miscarriage rates, which included 3815 women undergoing FET from 18 randomized control trials (RCTs), failed to provide sufficient evidence in favor of any specific protocol of endometrial preparation [17]. 

One of the most accurate studies published so far, a prospective multicenter RCT on 959 patients, did not show substantial differences between NC/m-NC or AC FETs, not only in terms of birth rate but also in terms of cost [18]. 

Given the above evidence, the artificial preparation of the endometrium has become quite popular worldwide as the first choice, considering its ease of handling and the possibility of using it in both ovulatory and anovulatory women [19]. Compared to NC/m-NC endometrial preparation, the AC protocol allows for clinic workloads to be better planned, avoids ETs during weekends, is easily organized even at a distance, and applies to all women independent of their menstrual regularity. As a matter of fact, AC endometrial preparation is also increasingly being used for women who wish to avoid repeated ovulation monitoring via ultrasound and for egg donation cycles.

However, in contrast to the prevailing notion, some recent studies suggest that different endometrial preparation protocols for FET might result in different outcomes, even in terms of pregnancy rates. High concentrations of circulating estradiol, which are reached during estrogen supplementation, have been associated with reduced success [20]. An early closure of the implantation window has been suggested as a potential mechanism behind this association [21]. Furthermore, some studies report an increased incidence of miscarriage after AC preparation, pointing to the significant role of the corpus luteum in preventing abortion [22]. Indeed, higher live birth rates for FETs performed during either a spontaneous ovulatory cycle or after the induction of ovulation have been reported [6,23]). 

In the present study, no differences were noted between the groups in terms of live births and miscarriages. While no conclusions can be drawn, given the limited number of observations, these data are in line with the notion that the endometrium is equally receptive in artificial vs. natural cycles, provided that sufficient estrogens and progesterone are available [14]. Whether specific methods of artificial endometrial preparation would be better than others in terms of pregnancy and miscarriage rates remains to be determined.

In recent times, AC FETs have been considered from a different perspective, i.e., the absence of a corpus luteum. As a matter of fact, the role of the corpus luteum goes beyond estrogen and progesterone secretion since it releases several other substances involved in the physiology of pregnancy. 

In early pregnancies conceived in the absence of a corpus luteum, circulating concentrations of both renin and prorenin are lower than in naturally conceived pregnancies [24]. The same is observed for relaxin, a molecule that plays a pivotal role in the maternal physiologic adaptation to pregnancy. Relaxin is actually involved in the regulation of cardiovascular and renal functions and in the control of plasma volume [11,25,26]. 

The lack of these factors would result in the maladaptation of the maternal cardiovascular system, resulting in increased risks of hypertensive disorders and PE.

As a further consideration, while adequate serum levels of both estradiol and progesterone are obtained during AC, the possibility exists that these hormones only reach subnormal concentrations in the endometrium. If this were the case, placentation anomalies could occur via the altered trophoblastic invasion of the decidua. The final consequence could be a suboptimal blood supply to the placenta, with abnormal decidualization and a higher risk of excessive trophoblast invasion [27,28]. Indeed, previous studies support the notion of an increased risk of abnormal placental insertion in artificially prepared FET cycles [6]. Of note, uterine surgery represents a risk factor for this pathology [29]. Obviously, the limited number of observations did not allow for statistical analysis. However, it should be stressed that whereas previous uterine surgeries were virtually limited to the NC/m-NC study group, the only cases (10%) of abnormal placentation were registered in the AC group.

We are aware that our study has some weakness. As already discussed, the number of pregnancies examined is limited. This factor has reduced the potential for further sub-analysis, for example, between NCs and m-NCs. We found no substantial differences in the two subgroups, although the limited numbers led us to exclude these results. We do not think that these two approaches can lead to different outcomes: they are similar in pathophysiological terms, since both involve the presence of the corpus luteum. Furthermore, there are all known limitations of retrospective studies, and most of the patients included were of Caucasian ethnicity. We advise caution in generalizing our data, especially regarding the PIH outcome, which we know is closely associated with ethnicity [30]. 

To our knowledge, this is the first study of its kind in an Italian population. In this country, from 2014 to 2020, thawing cycles increased from 11,000 to more than 20,000 (Italian registry data). The use of thawing cycles has spread not only in Italy but throughout the world. For this reason, increasing the available evidence regarding the outcomes of pregnancies obtained via different endometrial preparation strategies is necessary. Italy also holds the record for the most advanced female age upon the birth of the first child (31.4 years, EUROSTAT). Since age alone represents one of the main risk factors for obstetric complications, adopting treatment strategies that can limit these risks, especially in IVF pregnancies, is pivotal. 

All treatments were performed in a single center, ensuring high homogeneity with respect to the choice of protocol according to the patients’ individual characteristics and lab processes. Furthermore, the management of all pregnancies and obstetric complications was uniform as it was performed within the same hospital (Sant’Anna of Turin), which represents the nation’s reference center for number of deliveries (more than 6000 per year) and is one of the largest maternal–fetal centers in Europe.

Some questions remain unanswered, leaving space for further research on this topic. First of all, it would be interesting to understand whether anti-inflammatory, anti-coagulant, or anti-aggregating molecules are able to reduce the impact of AC preparation on placentation and/or hypertensive disorders. Strong evidence suggests that the administration of low-dose aspirin, initiated in the first trimester, significantly reduces the rate of preeclampsia [30,31]. Although aspirin does not seem to be able to reduce the risks related to AC preparation in our study, further large, prospective randomized trials should be performed. At the same time, it would be interesting to investigate whether different methods of progesterone administration in an AC would lead to overlapping results. For these reasons, future studies exploring the efficiency of different molecules (dydrogesterone), routes of administration, and optimal serum progesterone levels in ACs are desirable.

In conclusion, with the limitation of its retrospective nature and the relatively low number of observations, our study encourages the use of a natural cycle for endometrial preparation. Even for women with chronic anovulation, the induction of ovulation via a low dose of gonadotropins or selective estrogen receptor modulators (clomiphene and letrozole) can be successfully used to ensure the presence of a natural corpus luteum able to orchestrate maternal adaptation to pregnancy, reserving the use of AC preparation only for cases of precocious ovulation failure treated via egg donation.

Growing evidence shows that pregnancies achieved after spontaneous/modified endometrial preparation for FET have more favorable outcomes. Further research studies are needed to identify strategies capable of improving pregnancy outcomes after AC-FET, as this regime is the only one applicable in patients with ovarian failure who are undergoing egg donation. 

## Figures and Tables

**Table 1:** Reproductive outcomes of the two groups of patients (natural or modified natural cycles and artificial cycles).

	NC/m-NC *n* = 239	AC *n* = 78	*p*
**Positive pregnancy test, *n* (%)**	103 (43.1)	40 (51.3)	0.2071
**Clinical pregnancy at US, *n* (%)**	91 (38.1)	35 (44.9)	0.2869
**Miscarriage < 12 w, *n* (%)**	11 (12.1)	5 (14.2)	0.6259
**Late abortion ≥ 12 w, *n* (%)**	1 (1.1)	0 (0)	>0.9999
**Ectopic, *n* (%)**	3 (3.3)	1 (2.8)	>0.9999
**Therapeutic pregnancy termination, *n* (%)**	1 (1.1)	1 (2.8)	0.5164
**Live birth, *n* (%)**	75 (31.4)	29 (37.2)	0.2760
**Singleton live birth, *n* (%)**	75 (31.4)	28 (35.9)	0.4596

**Table 2:** Patient characteristics and treatments of the women who reached delivery (natural or modified natural cycle vs. artificial cycle).

	NC/m-NC *n* = 75	AC *n* = 28	*p*
**Maternal age at embryo freezing, mean (SD)**	34.1 (4.2)	33.7 (3.5)	0.6096
**Maternal age at embryo thawing, mean (SD)**	34.9 (4)	34.4 (3.8)	0.4817
**Maternal age at embryo thawing, *n* (%)**			0.5076
≤34	35 (47.3)	17 (60.7)	
35–39	28 (37.8)	8 (28.6)	
≥40	11 (14.9)	3 (10.7)	
**Primary infertility, *n* (%)**	57 (76)	24 (85.7)	0.2845
**Race, *n* (%)**			0.9155
Caucasian	68 (90.7)	26 (92.9)	
Asian	3 (4)	0 (0)	
African	2 (2.7)	1 (3.6)	
South American	2 (2.7)	1 (3.6)	
**Smoking habit, *n* (%)**	8 (14.3)	2 (8.3)	0.7149
**Maternal BMI, mean (SD)**	22.8 (3.4)	23 (4.9)	0.8320
**Maternal BMI, *n* (%)**			0.1823
<18.5	2 (3)	3 (12)	
18.5–24.9	49 (74.2)	14 (56)	
25–29.9	11 (16.7)	5 (20)	
≥30	4 (6.1)	3 (12)	
**Years of infertility, *n* (%)**			0.0237
1–2	31 (43.7)	7 (28)	
3–4	23 (32.4)	16 (64)	
≥5	17 (23.9)	2 (8)	
**Cause of infertility, *n* (%)**			0.0061
Oligo/anovulation	4 (5.5)	9 (33.3)	
Endometriosis	3 (4.1)	0 (0)	
Male factor	32 (43.8)	8 (29.6)	
Tubal factor	5 (6.8)	0 (0)	
Unexplained	16 (21.9)	8 (29.6)	
Mixed	13 (17.8)	2 (7.4)	
**Antral Follicle Count, mean (SD)**	18.2 (7.5)	27.5 (11.9)	<0.001
AMH (ng/mL), mean (SD)	5 (7)	10.6 (9.2)	0.0089
**Sperm origin, *n* (%)**			0.3741
Fresh	67 (89.3)	27 (100)	
Frozen	4 (5.3)	0 (0)	
Surgical	4 (5.3)	0 (0)	
**ART method, *n* (%)**			0.1253
IVF	23 (30.7)	5 (17.9)	
ICSI	44 (58.7)	16 (57.1)	
Combined	8 (10.7)	6 (21.4)	
**Culture until blastocyst stage (day 5–6) *n* (%)**	73 (97.3)	27 (96.4)	>0.9999
**OHSS, *n* (%)**	5 (7.1)	1 (3.7)	>0.9999
**Freeze-all strategy, *n* (%)**	36 (48)	25 (92.6)	<0.001
**Single embryo transfer, *n* (%)**	73 (97.3)	27 (96.4)	>0.9999
**Endometrial thickness at ET (mm), mean (SD)**	8.1 (1.7)	8.5 (1.9)	0.3292
**Pre-existing comorbidity, *n* (%)**			
Disthyroidism	22 (29.3)	8 (28.6)	0.9396
Hyperprolactinemia	11 (14.7)	0 (0)	0.0329
Hypertension	0 (0)	0 (0)	
Obesity	4 (5.3)	3 (10.7)	0.3862
Other	19 (25.3)	4 (14.3)	0.2310
**Autoimmune antibodies, *n* (%)**	14 (18.9)	6 (21.4)	0.7757
**Gynecological comorbidity, *n* (%)**			
PCOS	4 (5.3)	10 (35.7)	<0.001
Hypothalamic amenorrhea	0 (0)	1 (3.6)	0.2718
Endometriosis	7 (9.3)	0 (0)	0.1852
Uterine fibromatosis	7 (9.3)	2 (7.1)	>0.9999
Previous PID	3 (4)	1 (3.6)	>0.9999
**Previous gynecological surgery, *n* (%)**			
Adnexal surgery	7 (9.3)	1 (3.6)	0.4421
Miomectomy	2 (2.7)	0 (0)	>0.9999
Polypectomy	8 (10.7)	1 (3.6)	0.4382
Endometriosis ablation	3 (4)	0 (0)	0.5606
Other	4 (5.3)	1 (3.6)	>0.9999
**Therapy during pregnancy, *n* (%)**			
Low-dose acetylsalicylic acid (ASA)	12 (18.5)	8 (30.8)	0.2002

**Table 3:** Obstetric outcomes of the two groups (natural or modified natural cycle and artificial cycle).

	NC/m-NC *n* = 75	AC *n* = 28	*p*
**Gestational age (weeks), median (Q1; Q3)**	39 + 3 (38; 40 + 6)	39 + 3 (38 + 6; 40 + 1)	0.9475
**Type of delivery, *n* (%)**			0.6159
Spontaneous vaginal delivery	45 (60)	15 (53.6)	
Operative vaginal delivery	13 (17.3)	4 (14.3)	
C-section	17 (22.7)	9 (32.1)	
**Labour induction, *n* (%)**	21 (31.8)	14 (53.8)	0.0500
**pPROM, *n* (%)**	3 (4)	2 (7.1)	0.6112
**Chorioamniotitis, *n* (%)**	3 (4)	0 (0)	0.5606
**PPH, *n* (%)**	12 (16)	12 (42.9)	0.0041
**OASIS, *n* (%)**	2 (2.7)	2 (7.1)	0.2975
**Manual placenta removal, *n* (%)**	1 (1.3)	1 (3.6)	0.4717
**Placenta weight, mean (SD)**	596.5 (136.7)	532.9 (104.5)	0.1163
**Placental insertion anomalies, *n* (%)**	0 (0)	3 (10.7)	0.0191
**HDP, *n* (%)**	4 (5.3)	5 (17.9)	0.0591
**PIH, *n* (%)**	3 (4)	5 (17.9)	0.0327
**PE, *n* (%)**	1 (1.3)	0 (0)	>0.9999
**GDM, *n* (%)**	9 (12)	7 (25)	0.1293
**Cholestasis, *n* (%)**	1 (1.3)	1 (3.6)	0.4717
**Hospitalization during pregnancy, *n* (%)**	14 (18.7)	8 (28.6)	0.2752

**Table 4:** Univariate logistic regression analysis for hypertensive disorders of pregnancy, pregnancy-induced hypertension, and placental insertion anomalies.

	NC/m-NC *n* = 75	AC *n* = 28	*p*	OR	IC 95%	aOR	IC 95%
**HDP, *n* (%)**	4 (5.3)	5 (17.9)	0.0591	3.86	0.955–15.594	2.34	0.502–10.872
**PIH, *n* (%)**	12 (16)	12 (42.9)	0.0041	3.94	1.492–10.389	4.38	1.507–12.747
**Placental insertion anomalies, *n* (%)**	0 (0)	3 (10.7)	0.0191	-	-	-	-

**Table 5:** Neonatal outcomes of the two groups (natural or modified natural cycle and artificial cycle).

	NC/m-NC *n* = 75	AC *n* = 28	*p*
**Male sex, *n*** **(%)**	38 (50.7)	14 (50)	0.9520
**Late preterm (32–37 w), *n*** **(%)**	6 (8)	5 (17.9)	0.1647
**Birthweight,** **mean (SD)**	3253.1 (557.3)	3119.1 (543.2)	0.2771
**LGA, *n*** **(%)**	7 (9.3)	2 (7.1)	>0.9999
**Macrosomia ≥ 4000 g, *n* (%)**	7 (9.3)	1 (3.6)	0.4421
**SGA, *n* (%)**	6 (8)	2 (7.1)	>0.9999
**IUGR ≥ 32 w, *n*** **(%)**	5 (6.7)	2 (7.1)	>0.9999
**APGAR ≤ 7 at 5′, *n* (%)**	2 (2.7)	0 (0)	>0.9999
**Arterial pH at birth, mean (SD)**	7.24 (0.9)	7.23 (0.1)	0.5160
**Neonatal hospitalization, *n*** **(%)**	4 (5.6)	3 (10.7)	0.4000

## Data Availability

The data presented in this study are available upon request from the corresponding author. The data are not publicly available due to IRB rules.
